# Association between hypothyroidism and metabolic syndrome in Qinghai, China

**DOI:** 10.3389/fendo.2024.1407996

**Published:** 2024-10-25

**Authors:** Xiaoxia Fan, Yongli Yao, Shengjun Chai, Beibei Wang, Yanling Xie, Yanping Jiang, Lijun Lin, Yanan Li, Peiyun Fan, Wei Luo, Shuqiong Wang, Kang Song, Lingling Zhao, Chunmei Cai

**Affiliations:** ^1^ Research Center for High Altitude Medicine, Qinghai University Medical College, Xining, Qinghai, China; ^2^ Key Laboratory of the Ministry of High Altitude Medicine, Qinghai University Medical College, Xining, Qinghai, China; ^3^ Key Laboratory of Applied Fundamentals of High Altitude Medicine, (Qinghai-Utah Joint Key Laboratory of Plateau Medicine), Qinghai University Medical College, Xining, Qinghai, China; ^4^ Laboratory for High Altitude Medicine of Qinghai Province, Qinghai University Medical College, Xining, Qinghai, China; ^5^ Department of Endocrinology, Qinghai Provincial People’s Hospital, Xining, Qinghai, China

**Keywords:** Qinghai province, hypothyroidism, epidemiology, metabolic syndrome, China

## Abstract

**Objective:**

To investigate the epidemiological characteristics of hypothyroidism in Qinghai Province, analyze its related influencing factors, establish the normal reference range of thyroid function, and explore the relationship between thyroid hormone (THs), thyroid stimulating hormone (TSH) and metabolic syndrome (MS) in Qinghai population within the normal range, so as to provide some scientific basis for the prevention and treatment of hypothyroidism in Qinghai Province.

**Methods:**

A total of 2790 residents aged 18 and over from Qinghai were selected through stratified cluster random sampling. Data were collected via questionnaires, physical examinations, and laboratory tests.

**Results:**

1. A total of 2628 eligible residents in Qinghai were included in this study, and the total prevalence of hypothyroidism was 30.25%, among which the prevalence of subclinical hypothyroidism was 29.22%, and the prevalence of clinical hypothyroidism was 1.03%. 2. The prevalence of hypothyroidism in women was significantly higher than that in men (36.69% vs 24.30%); smoking and drinking were risk factors for hypothyroidism. 3. In the excluded subjects, 1544 were abnormal thyroid ultrasound, abnormal thyroid function and/or positive thyroid autoantibodies, and the remaining 1084 were reference populations. According to the reference population data, the 95% reference ranges of TSH, FT4, FT3 were 0.43-5.51 mIU/L, 11.0-20.4 pmol/L, 3.63-5.73 pmol/L, respectively. 4. In the normal thyroid function population in Qinghai, MS and its related components were positively correlated with FT3 and FT4 levels, but not significantly correlated with TSH levels.

**Conclusion:**

1. The prevalence of hypothyroidism in adults in Qinghai is relatively high, accounting for about one-thirtieth of the total population. Smoking and drinking have a certain impact on the incidence of hypothyroidism. 2. It provides a reference range for the diagnosis of thyroid diseases in Qinghai province, which is different from that of reagent suppliers, and has certain promotion significance in the western region. 3. MS and its related components are positively correlated with FT3 and FT4 levels, but not with TSH levels in people with normal thyroid function in Qinghai. Early thyroid function screening is of great significance for the prevention of MS.

## Highlights

This study investigated the epidemiological characteristics of hypothyroidism including clinical hypothyroidism and subclinical hypothyroidism in Qinghai population, analyzed the related influencing factors, and established the normal reference range of thyroid function. The relationship between thyroid hormone (THs), thyroid stimulating hormone (TSH) and metabolic syndrome (MS) in the normal range of Qinghai population was discussed, so as to provide part of the scientific basis for the prevention and treatment of hypothyroidism and saving related medical expenses in Qinghai province. It is helpful for the diagnosis of thyroid diseases and the prevention and control of MS and related diseases.

In this study, stratified cluster random sampling and voluntary participation were used to collect and measure a number of relevant clinical indicators of all subjects through laboratory examination and questionnaire survey. The epidemiological characteristics and related risk factors of hypothyroidism of adult permanent residents in Qinghai Province were analyzed, and the reference range of normal thyroid hormone levels in Qinghai population was established. The correlation between thyroid hormone levels and MS and its components in the normal range was analyzed by cross-sectional study, and the following conclusions were drawn:

1. A total of 2628 eligible Qinghai residents were included in this study. The total prevalence of hypothyroidism was 30.3%, of which the prevalence of subclinical hypothyroidism was 29.2%, and the prevalence of clinical hypothyroidism was 1.0%. The prevalence of hypothyroidism in women was significantly higher than that in men (1.4% vs 0.7%). Women are susceptible to hypothyroidism. Smoking and drinking are risk factors for hypothyroidism.
2. After excluding 1544 subjects with abnormal thyroid ultrasound, abnormal thyroid function and/or positive thyroid autoantibodies, 1084 subjects were left as the reference value population. The 95% reference intervals of TSH, Free thyroxine (FT4) and Free triiodothyronine (FT3) were 0.43-5.51mIU/L, 11.0-20.4pmol/L and 3.63-5.73pmol/L, respectively.
3. The levels of FT3, FT4 and TSH in metabolic syndrome patients with normal thyroid function (blood pressure, blood lipid, body mass index, etc.) are related to the levels of FT3, FT4 and TSH.

## 1 Introduction

The prevalence of hypothyroidism is increasing, and more and more studies have reported that it is related to the occurrence and progression of a variety of diseases (1). Early detection and early intervention of hypothyroidism has a very positive significance for the protection of people’s systemic health. Understanding the epidemiological characteristics and influencing factors of hypothyroidism in a region can provide reference for the development of prevention and treatment strategies for the disease. The accurate diagnosis of thyroid diseases depends on the accurate reference range (RIs) of thyroid function. Previous studies have shown that RIs of different regions, races and ages vary greatly (2). However, the current situation is that most laboratories in China usually refer to RIs provided by reagent suppliers. There is a certain overlap between MS and thyroid dysfunction in the pathogenesis and disease process, and the prevalence of MS, like hypothyroidism, has increased significantly worldwide in recent years. Nowadays, there are more studies on abnormal thyroid function and MS and the conclusions are relatively clear. However, there are few studies on the relationship between serum thyroid levels (including FT3, FT4 and TSH) in the normal range and MS and its components. Qinghai Province is located in the northeast of the Qinghai-Tibet Plateau on the top of the world, and belongs to a high-altitude area, with an altitude of 1800cm-4300cm. It has poor hypoxia, poor oxygenation, higher blood lipids, and more complications. Neither the epidemiological survey of hypothyroidism nor the establishment of reference ranges of thyroid function in normal population are available. The impact of different metabolic factors on the occurrence and development of thyroid function in this region needs to be further clarified by basic and clinical trials. Based on the above research background, this project is based on the Qinghai area to investigate the epidemiological characteristics of hypothyroidism and subclinical hypothyroidism in specific populations, analyze the related influencing factors, establish the normal reference range of thyroid function, and discuss the relationship between THs, TSH and MS in the normal range of Qinghai population. The status of hypothyroidism in the Qinghai population was assessed to obtain complete data on the current correlation between hypothyroidism and MS.

## 2 Materials and methods

### 2.1 Study participants

This study is part of the TIDE (3) project of the First Hospital of China Medical University. TIDE refers to the 2010 Chinese national census data to determine the age and gender composition of each community and the urban-rural ratio. We planned to include a total survey population of 2,650 people. Inclusion criteria: Community residents aged>18 years, permanent residents who have lived in the region for more than 5 years. Exclusion criteria: pregnancy; severe liver and kidney disease; recent use of hormone drugs (except thyroid hormone replacement therapy). This study was approved by the Medical Research Ethics Committee of the First Affiliated Hospital of China Medical University. All participants provided written informed consent after receiving a thorough explanation of the research procedures.

### 2.2 Survey process

One week before the on-site investigation, the members of the research group publicized the project to the selected population, issued and signed the informed consent form. The participants were instructed to fast after dinner one day before the on-site investigation, and to ensure that they were fasted for at least 10 hours on the day of the on-site investigation. After arriving at the site on the day of the field investigation, fasting blood samples were drawn, and then 75g oral glucose tolerance test (OGTT) was performed for all participants without a history of diabetes, and steamed bread meal OGTT was performed for those with a history of diabetes (about 2 2 steamed bread, equivalent to 80g carbohydrate). All participants should drink the glucose water or eat the steamed bread meal within 5 minutes, and draw elbow vein blood again 30 minutes and 120 minutes later. (Note: The participants should not take insulin, hypoglycemic drugs and other drugs that affect glucose metabolism in the morning of the glucose tolerance test.) All blood samples were sent to the laboratory department of Qinghai Provincial People’s Hospital. Fasting blood samples were used to detect thyroid function and various biochemical indicators, and 30 minutes and 120 minutes of blood samples were used to detect postprandial blood glucose and insulin at corresponding times. While waiting for postprandial blood sampling, participants were organized to collect height, weight, blood pressure, waist circumference, hip circumference and questionnaire data.

### 2.3 Survey methods

#### 2.3.1 Questionnaire survey

A unified designed questionnaire was used to collect the general information of the research objects. All investigators were medical personnel with uniform training. The questionnaire mainly included the following aspects:

1. Demographic characteristics (age, gender, ethnicity, marital status, education level, occupation);
2. Medical history and medication history (history of diabetes, family history of diabetes, history of hypertension, history of dyslipidemia, coronary History of heart disease, stroke, liver and kidney disease, malignant tumor disease and related conditions, family history of thyroid disease, history of thyroid disease, diagnosis and treatment, etc.).
3. lifestyle (smoking, drinking, dietary taste, seafood intake, physical labor, sports) Exercise, etc.).

#### 2.3.2 Classification criteria

1. Classification of education level: never going to school, primary school and junior high school, senior high school/technical school/technical secondary school, college degree or above.
2. Smoking status: smoking (including current smoking and former smoking who have quit) and Non-smoking.
3. Drinking status: divided into drinking (including current drinking and former drinking and ex-drinking) and non-drinking.
4. Eating food taste (salt habit):
  1= severe (> 10 g/person/day)
  2= moderate (5-10 g/person/day)
  3= mild (less than 5 g/person/day)
  Reference: toothpaste cap =5 g beer bottle cap =10 g
5. Eating seafood habits (such as kelp, laver, seafood, etc.):
  1 = often (weekly)
  2 = Occasionally (monthly)
  3 = Not eating
6. Annual household income:
  1= less than 5000 yuan
  2 = 50,000-10,000 yuan
  3 = 10,000 to 30,000 yuan
  4 = 30,000 to 50,000 yuan
  5 = 50,000-100,000 yuan
  6= more than 100,000 yuan

#### 2.3.3 Laboratory tests

The fasting blood, 30min and 120min after eating blood samples were collected from the elbow vein of the participants by trained professional medical staff. The blood samples were placed in a refrigerated incubator at room temperature for no more than 2 hours, then centrifuged at 3000r/min for 10 minutes, and the separated serum samples were frozen and stored in the refrigerator at -20°C. They were sent to the laboratory department of Qinghai Provincial People’s Hospital within 3 days for the determination of relevant clinical indicators. Cobas from Roche, Germany was used Fasting plasma glucose (FPG), fasting insulin (FINS), 30min postprandial plasma glucose (PG 30 min), 30min postprandial insulin (INS 30 min), 2h postprandial plasma glucose (PG 2h), 2h postprandial insulin (INS 2h), triglyceride (TG), total cholesterol (TC), High density apolipoprotein (HDL-C), and low density apolipoprotein (LDL-C). FT3, FT4, TSH, TPO Ab, TR Ab and Tg Ab were measured by chemiluminescence immunoassay using a Cobas 601 analyzer (Roche Diagnostics, Switzerland) at the central laboratory in Shenyang with the same way.

### 2.4 Physical examination

The measurements were completed by trained medical staff in accordance with the recommended methods in the Handbook of Cardiovascular Epidemiological Investigation Methods. Main measurements and methods were as follows:

1. Height: the subject took off his shoes and hat and stood barefooted, his heels were tight, his eyes were looking straight ahead, and the reading was taken.
  The height of the tester was counted to l cm.
2. Body weight: the subjects emptied their skin, wore only thin clothes, took off their shoes and hats, and stood on their body weight on an empty stomach.
  Meter (corrected) disc center, meter reading accurate to l kg.
3. Waist circumference: the subjects stood with their feet 25-30cm apart, and their weight was evenly distributed. With a scale tape, the waist circumference was measured at the end of expiration around the abdomen at the midpoint of the line between the anterior superior iliac spine and the lower edge of the 12th rib (the horizontal line of the umbilicum), and the count was accurate to l cm.
4. Hip circumference: the subject’s legs stand together, the two arms naturally drop, and the tape measure is placed horizontally in front of the pubic bone. The hip circumference was calculated by circling the symphysis and the most convexity of the gluteus maximus in the back, and the count was accurate to the l cm.
5. Blood pressure: the subjects should not smoke, drink coffee, tea and empty the bladder within 30 minutes, and rest at least 5 minutes. The brachial artery blood pressure of the right upper arm was measured with a unified calibration electronic sphygmomanometer in a sitting position. The upper arm was at the same level as the heart, and the measurement was repeated at an interval of 1-2 minutes for a total of 3 times.

### 2.5 Definition of disease

#### 2.5.1 Diagnostic criteria for metabolic syndrome

According to the diagnostic criteria of MS recommended by the Joint Committee for the Development of Guidelines for the Prevention and Treatment of Dyslipidemia in Chinese Adults (JCDCG) in 2022 (4), the specific diagnostic criteria are shown in **Table 1**.

**Table 1:** Diagnostic criteria for MS.

Disease	Diagnostic criteria
Abdominal obesity	Waist circumference >90cm in men and >85cm in women
Hyperglycemia	Fasting blood glucose >6.1 mmol/L or 2h after OGTT blood glucose >7.8 mmol/L and/or diagnosed and treated with diabetes
Hypertension	Blood pressure >130/85 mmHg and/or hypertension was confirmed and treated.
Elevated riglycerides	Fasting TG>1.70 mmol/L
Decreased HDL cholesterol	Fasting HDL-C<1.04 mmol/L.

Three or more of these are diagnostic.

#### 2.5.2 Thyroid function status was defined

Thyroid function was classified according to the reference range of normal THs level established in this study (**Table 2**).

**Table 2:** Diagnostic criteria for thyroid diseases and classification criteria.

Thyroid disease	Diagnostic criteria
Clinical hypothyroidism	TSH>4.2mIU/L;FT4<12pmol/L
Subclinical hypothyroidism (SCH)	TSH>4.2mIU/L;FT4 12-22pmol/L
TPOAb positive	TPOAb>34 IU/ml
TgAb positive	TgAb>115 IU/ml

### 2.6 Statistical methods

The measurement data with normal distribution were expressed as mean ± standard deviation ( $\bar{x}$ ± s), the measurement data with non-normal distribution were expressed as median (interquartile range), and the count data were expressed as frequency or percentage. The measurement data with normal distribution were compared between the two groups by independent sample t test, and the comparison among multiple groups was performed by analysis of variance, and LSD method was used for pairwise comparison. Non-normal distribution data were transformed to normal distribution with logarithmic transformation, and then ANOVA was performed between groups. If the measurement data could not meet the normal distribution after log transformation, rank sum test was used. Pearson chi-square test was used to compare the count data between groups. Multiple linear regression analysis and multivariate logistic regression analysis were used for correlation analysis. Statistical significance and significant statistical significance were defined as P< 0.05 and P< 0.01, respectively.

## 3 Research results

### 3.1 General situation of survey objects

A total of 2790 participants were selected for questionnaire survey, physical examination and laboratory tests. Among them, 72 participants with incomplete key information collection and 90 participants with incomplete laboratory tests were excluded, and 2628 participants were finally included in this study, including 1358 males (51.67%) and 1270 females (48.33%). The ratio of female to male was 1.07:1, and the average age was 43.37 ± 5.37 years. 1374 (52.28%) were urban residents and 1254 (47.72%) were rural residents (**Table 3**).

**Table 3:** General characteristics of 2628 subjects.

Variables	N	Proportion of proportion(%)
Number	2628	100
Location		
Urban	1374	52.28
Rural	1254	47.72
Gender		
Male	1358	51.67
Female	1270	48.33
Age (years)		
18-	635	24.16
30-	547	20.81
40-	569	21.65
50-	424	16.13
60-	263	10.00
≥70	190	7.23

### 3.2 Prevalence of hypothyroidism by gender and age

The overall prevalence of hypothyroidism in Qinghai population was 30.25% (796/2628). The prevalence of subclinical hypothyroidism was 29.22% (768/2628), which was significantly higher than that of clinical hypothyroidism 1.03% (28/2628). The prevalence of clinical hypothyroidism, subclinical hypothyroidism and hypothyroidism (including clinical hypothyroidism and subclinical hypothyroidism) were significantly higher in women than in men (1.42%vs0.73%, 35.28%vs23.56%, 36.69%vs24.30%). The difference between subclinical hypothyroidism and hypothyroidism was statistically significant. The prevalence of clinical hypothyroidism was the highest in the ≥70 age group (2.63%) and the lowest in the 18-year age group (0.0%). The difference between the age groups was statistically significant (P=0.012). The prevalence of subclinical hypothyroidism was the highest in the 60-year-old group (40.68%), and the lowest in the 30-year-old group (20.66%). The highest prevalence of hypothyroidism was 41.83% in the 60-year-old group, and the lowest prevalence was 21.57% in the 30-year-old group. The difference between the age groups was statistically significant (P =0.000). (**Table 4**).

**Table 4:** Comparison of the prevalence of hypothyroidism by gender and age (%).

Variables	The number of survey	Clinical hypothyroidism	Subclinical hypothyroidism	Hypothyroidism
	N(%)	N(%)	N(%)	N(%)
Gender				
Male	1358(51.67)	9(0.66)	320(23.56)	330(24.30)
Female	1270(48.33)	18(1.42)	448(35.28)	466(36.69)
χ2		3.667	43.365	47.187
P		0.042	<0.001	<0.001
Age (years)				
18-	636(24.20)	0(0)	137(25.56)	137(25.56)
30-	547(20.82)	5(0.91)	113(20.66)	118(21.57)
40-	568(21.61)	10(1.76)	181(31.87)	191(33.63)
50-	424(16.14)	4(0.94)	163(38.44)	167(39.39)
60-	263(10.01)	3(1.14)	107(40.68)	110(41.83)
≥70	190(7.23)	5(2.63)	67(35.26)	72(37.89)
χ2		14.544	76.804	79.944
P		0.013	<0.001	<0.001
合计	2628	28(1.07)	768(29.22)	796(30.29)

### 3.3 Prevalence of hypothyroidism in different age groups of male

The prevalence of male clinical hypothyroidism was the highest in the ≥70 years group (2.11%), and the lowest in the 18-year group (0%). There was no significant difference in the prevalence of clinical hypothyroidism between different age groups (P=0.013). The prevalence of subclinical hypothyroidism in males was the highest in the 60-year-old group (37.31%) and the lowest in the 30-year-old group (15.02%). There were significant differences in the prevalence of subclinical hypothyroidism among different age groups (P=0.000). The prevalence of male hypothyroidism was the highest in the 70-year-old group (36.84%), and the lowest in the 18-year-old group (15.50%). There were significant differences in the prevalence of hypothyroidism among different age groups (P=0.000) (**Table 5**)

**Table 5:** Comparison of prevalence of hypothyroidism among different age groups in men (%).

Age (years)	The number of survey	Clinical hypothyroidism	Subclinical hypothyroidism	Hypothyroidism
	N(%)	N(%)	N(%)	N(%)
18-	329(24.23)	0(0.0)	51(15.50)	51(15.50)
30-	273(20.10)	2(0.73)	41(15.02)	43(15.75)
40-	292(21.50)	3(1.03)	71(24.32)	74(25.34)
50-	235(17.30)	1(0.43)	74(31.48)	75(31.91)
60-	134(9.87)	1(0.75)	50(37.31)	51(38.06)
≥70	95(7.0)	2(2.11)	33(34.74)	35(36.84)
χ2		14.544	51.671	53.32
P		0.013	0.000	0.000

### 3.4 Prevalence of hypothyroidism in different age groups of female

The prevalence of clinical hypothyroidism in women was the highest in the ≥70 years group (3.16%), and the lowest in the 18-29 years group (0.0%). There was no significant difference in the prevalence of clinical hypothyroidism among different age groups (P=0.101). The prevalence of subclinical hypothyroidism in women was the highest in the 50-year old group (47.09%), and the lowest in the 30-year old group (26.28%). There were significant differences in the prevalence of subclinical hypothyroidism among different age groups (P< 0.001). The prevalence of hypothyroidism in women was the highest in the 50-year old group (48.68%), and the lowest in the 40-year old group (6.52%). There were significant differences in the prevalence of hypothyroidism among different age groups (P =0.000) (**Table 6**).

**Table 6:** Comparison of prevalence of hypothyroidism among different age groups in women (%).

Age (years)	The number of survey	Clinical hypothyroidism	Subclinical hypothyroidism	Hypothyroidism
	(%)	N(%)	N(%)	N(%)
18-	307(24.17)	0(0.0)	86(28.01)	86(28.01)
30-	274(21.57)	3(1.09)	72(26.28)	75(27.37)
40-	276(21.73)	7(2.54)	110(39.86)	18(6.52)
50-	189(14.88)	3(1.59)	89(47.09)	92(48.68)
60-	129(10.16)	2(1.55)	57(44.19)	59(45.74)
≥70	95(7.48)	3(3.16)	34(35.79)	37(38.95)
χ2		9.206	35.395	56.19
P		0.101	<0.001	<0.001

### 3.5 Comparison of prevalence of hypothyroidism in different lifestyles

In this study, there was no significant difference in the prevalence of clinical hypothyroidism among people with different education levels, smoking status, salt intake habits, seafood intake habits, and annual household income (P > 0.05). There was no significant difference in the prevalence of subclinical hypothyroidism and the prevalence of hypothyroidism between the habits of salt intake and seafood intake (P > 0.05), but there were significant differences in the prevalence of subclinical hypothyroidism and hypothyroidism among people with different education levels, smoking habits, and different annual household incomes (P< 0.05). The prevalence of clinical hypothyroidism and subclinical hypothyroidism in smokers were significantly higher than those in non-smokers (χ^2^ = 28.354, P = 0.000; χ^2^ = 27.627, P = 0.000; χ^2^ = 18.354, P = 0.000). The prevalence of clinical hypothyroidism, subclinical hypothyroidism and hypothyroidism in drinkers were significantly higher than those in non-drinkers (χ^2^ = 11.54, P=0.003; χ^2^ = 6.122, P=0.046; χ^2^ = 9.532, P=0.017). The prevalence of hypothyroidism was 13.96% in primary and junior high schools, and 9.98% in senior high schools/technical schools/technical secondary schools. There were significant differences in the prevalence of subclinical hypothyroidism and hypothyroidism among patients with different annual incomes (χ^2^ = 20.524, P=0.000; χ^2^ = 23.052, P=0.000) (**Table 7**).

**Table 7:** Comparison of Prevalence of hypothyroidism by lifestyle (%).

Variables	Group	Clinical hypothyroidism	Subclinical hypothyroidism	Hypothyroidism
		N(%)	N(%)	N(%)
Education level	Never going to school	4(1.42)	98(8.33)	102(3.88)
	Primary school and junior high school	15(1.09)	352(7.00)	367(13.96)
	Senior high school/technical school/technical secondary school	4(1.42)	163(9.98)	167(6.35)
	College degree or above	4(1.42)	155(7.28)	159(6.05)
Statistics	χ2	6.156	47.287	48.157
	P	0.291	0.000	0.000
Smoking status	Smoking	6(0.22)	165(6.28)	171(6.51)
	Non-smoking	21(0.80)	603(22.95)	624(23.74)
Statistics	χ2	0.560	27.627	28.354
	P	0.302	<0.001	<0.001
Alcohol Consumption status	Drinking	20(0.76)	456(17.35)	476(18.11)
	Non-drinking	7(0.27)	312(11.87)	319(12.14)
Statistics	χ2	11.54	6.122	9.532
	P	0.003	0.046	0.017
Salt intake habits	Severe	7(0.27%)	128(4.87%)	135(5.14%)
	moderate	19(0.72%)	546(20.8%)	565(21.50%)
	mild	1(0.04%)	94(3.6%)	95(3.61%)
Statistics	χ2	2.253	3.086	3.465
	P	0.324	0.214	0.229
Seafood intake habits	Severe	7(0.27)	192(7.31)	49(7.57)
	moderate	19(0.72)	409(15.56)	428(16.29)
	mild	1(0.04)	167(6.35)	168(6.39)
Statistics	χ2	0.114	0.205	0.198
	P	0.624	0.471	0.504
Income per year	-5000	1(0.04)	8(0.30)	0(0.00)
	5000-10000 yuan	2(0.08)	57(2.17)	59(2.25)
	10000-30000 yuan	4(0.15)	225(8.56)	229(8.71)
	30000-50000 yuan	15(0.57)	313(11.91)	328(12.5)
	50000-100000 yuan	4(1.47)	134(5.10)	138(5.25)
	More than100000 yuan	1(0.04)	31(1.18)	32(1.22)
Statistics	χ2	3.994	20.524	23.052
	P	0.550	0.001	0.000

### 3.6 Multivariate Logistic regression analysis of lifestyle and incidence of hypothyroidism

With or without hypothyroidism as the dependent variable, and education level, smoking status, salt intake habit, and annual household income as the independent variables, the variable assignment table is shown in (**Table 8**). Multivariate Logistic regression analysis showed that smoking (OR: 2.254, 95%CI: 1.107-4.591, P=0.025), drinking (OR: 1.218, 95%CI: 1.127-1.393, P=0.037) were the risk factors for hypothyroidism. Education level, salt intake habit, seafood intake habit, and household income were not found to be associated with hypothyroidism (**Tables 8**, **9**).

**Table 8:** Description of various research factors and quantitative methods.

Variables	Study factors	Code	Unit of quantification
Dependent variable	Prevalence of hypothyroidism	Y	0= no hypothyroidism 1= hypothyroidism
Independent variable	Degree of education	X1	0= never attended school 1= primary and junior high school
	Smoking status	X2	2= high school/technical school/technical secondary school
	Alcohol Consumption status	X3	3= college/university degree or above
	Salt intake habits	X4	0= smoking 1= no smoking
	Eating seafood habits	X5	0= alcohol consumption 1= no alcohol consumption
	Annual household income	X7	0= mild 1= moderate 2= severe

**Table 9:** Results of multivariate Logistic regression analysis of the incidence of hypothyroidism.

Study FACTORS	B	SE	P	OR	95%CI
Smoking status	0.813	0.363	0.025	2.254	1.107-4.591
Alcohol Consumption status	0.285		0.037	1.218	1.127~1.393

### 3.7 Reference range of normal thyroid hormone levels in Qinghai population and comparison of thyroid hormones in different age and gender

A total of 2628 subjects were included in this study. After excluding 1544 subjects with abnormal thyroid ultrasound, abnormal thyroid function and/or positive thyroid autoantibodies, 1084 subjects were used as the reference population. The 95% reference intervals of TSH, FT4 and FT3 in the reference population were 0.43-5.51mIU/L, 11.0-20.4pmol/L and 3.63-5.73pmol/L, respectively. The comparison of the difference of thyroid hormone (THs) levels in different age groups showed that the mean values of TSH in 51-60 years old and 61-70 years old were lower than those in 18-30 years old group, while the mean values of TSH in ≥70 years old group were higher than those in 18-30 years old group, and the differences were statistically significant (t=-1.45, P=0.012; t=-2.43, P=0.009; t=-2.04, P=0.007). There was no significant difference in FT3 and FT4 among different age groups (P > 0.05). The difference of THs between different genders in the reference population indicated that the TSH level of males was lower than that of females (t=-2.595, P=0.010), and FT3 and FT4 of males were higher than those of females (t=4.911, P=0.003, t=7.758, P =0.000), and the difference was statistically significant (**Table 10**).

**Table 10:** Reference range of normal thyroid hormone levels in Qinghai population and comparison of differences in different age and gender.

Grouping		N	TSH(mIU/L)	FT4(pmol/L)	FT3(pmol/L)
Age	18-29(ref.)	152	1.60(0.72~3.87)	16.1(12.6~19.5)	4.89(4.00~5.77)
	30-39	227	1.58(0.42~5.57)	16.0(11.2~20.7)	4.76(3.71~5.81)
	40-49	310	1.56(0.45~5.41)	15.6(10.7~20.5)	4.71(3.68~5.74)
	50-59	181	1.50(0.45~5.41)*	15.8(10.8~20.8)	4.70(3.53~5.86)
	60-69	123	1.49(0.39~5.68)*	15.5(11.2~19.9)	4.65(3.76~5.50)
	≥70	91	1.65(0.52~5.20)*	15.6(11.2~19.9)	4.66(3.45~5.18)
Gender	Male(ref.)	623	1.38(0.44~4.33)	16.3(11.4~21.1)	4.88(3.94~5.81)
	Female	461	1.69(0.43~6.53)#	15.3(10.9~19.7)#	4.51(3.48~5.54)#
Total		1084	1.54(0.43~5.51)	15.7(11.0~20.4)	4.68(3.63~5.73)

*indicates P< 0.05 compared with the age group of 18-29 years; # indicates P< 0.05 compared with the male group.

### 3.8 Cross-sectional comparison of normal thyroid function and metabolic syndrome in Qinghai population

#### 3.8.1 Comparison of THs levels between normal population and MS population with normal thyroid function

According to the reference range of thyroid hormone (THs) levels established in this study, 1327 subjects with normal thyroid function were selected, of which 929 subjects without metabolic syndrome (MS) were selected as normal population, including 376 males and 553 females. There were 398 participants with MS, including 211 males and 187 females. After adjusting for age and gender, the levels of FT3 and FT4 in the MS population were significantly higher than those in the normal population (P< 0.05), while the level of TSH was not statistically different (P=0.107). After further adjustment for BMI, there was no significant difference in FT3 level between the two groups (P=0.519), but FT4 level was still significantly different (P< 0.001) (**Table 11**).

**Table 11:** Comparison of THs levels between normal population and MS population with normal thyroid function.

Indicators	Normal population	MS (n=398)	P	P’
Age (years)	41.7 ± 13.0	50.3 ± 12.3	<0.001	<0.001
Gender (% male)	40.47%	53.02%	<0.001	–
BMI(kg/m^2^)	22.9 ± 2.9	26.4 ± 3.0	<0.001	–
FT3(pmol/L)	5.04 ± 0.60	5.09 ± 0.55	0.004	0.519
FT4(pmol/L)	15.9 ± 1.89	17.5 ± 1.93	<0.001	<0.001
TSH(mIU/L)	1.63(1.22)	1.56(1.16)	0.107	0.451

Measurement data were compared by analysis of covariance, P was corrected for gender and age, P ‘was further corrected for BMI, and enumeration data were compared by Pearson chi-square test.

#### 3.8.2 Correlation analysis of the incidence of metabolic syndrome and its related components with thyroid hormone levels in normal thyroid function

Logistic regression was used to calculate the odds ratio (OR) and 95% confidence interval (CI) of the second quantile (T2) and the third quantile (T3) of FT3, FT4 and TSH, respectively, with MS and its related components as dependent variables and the first quantile (T1) of FT3, FT4 and TSH as reference. Gender, age, smoking history and drinking history were included in the equation and substituted into the regression equation by the entry method. The results showed that: With the increase of FT3 level, the incidence and risk of MS and its components showed an increasing trend, except for hyperglycemia. The OR and 95%CI of MS, abdominal obesity, hypertension, high TG and low HDL-C in the third quartile of FT3 were 1.94 (1.45-2.58), 2.33 (1.80-3.02), 1.49 (1.17-1.90), 1.61 (1.25-2.08) and 1.40 (1.04-1.8), respectively 8), the results were statistically significant (P< 0.05). With the increase of FT4 level, the incidence and risk of MS and its components showed an increasing trend. The OR and 95%CI of MS, abdominal obesity, hyperglycemia, hypertension, high TG and low HDL-C in the third quartile of FT4 were 2.48 (1.93-3.17), 1.64 (1.32-2.03), 1.71 (1.34-2.17), 1.66 (1.35-2.05) and 2.28 (1.83), respectively -2.85), 1.57 (1.22-2.00), the results were statistically significant (P< 0.05); There was no significant difference in the incidence and risk of MS among the tertiles of TSH, but the risk of each component of MS was different with the change of TSH. Compared with the first quartile of TSH, the incidence and risk of abdominal obesity, high TG and low HDL-C were the highest in the third quartile, with OR and 95%CI of 1.28 (1.04-1.58), 1.38 (1.12-1.70) and 1.36 (1.07-1.74), respectively (P< 0.05). However, the incidence and risk of hyperglycemia showed a decreasing trend with the increase of TSH, and the third quartile OR and 95%CI were 0.74 (0.59-0.93) (P< 0.05). Different from the other components, the incidence and risk of hypertension were lowest in the second quartile of TSH. OR and 95%CI were 0.78 (0.064-0.93) (P< 0.05) (**Table 12**).

**Table 12:** Logistic regression analysis of the incidence of MS and its related components and THs levels in the context of normal thyroid function.

The project	Group	FT3		FT4		TSH	
		%	ORs (95%CI)	%	ORs (95%CI)	%	ORs (95%CI)
MS	T1	18.4	1 (Ref.)	16.4	1 (Ref.)	23.1	1 (Ref.)
	T2	24.7	1.58 (1.23~2.04)*	21.0	1.43 (1.11~1.84)*	22.8	1.20 (0.95~1.52)
	T3	26.1	1.94 (1.45~2.58)*	31.8	2.48 (1.93~3.17)*	23.3	1.26 (0.99~1.59)
Abdominal obesity	T1	24.9	1 (Ref.)	26.6	1 (Ref.)	30.2	1 (Ref.)
	T2	31.0	1.51 (1.21~1.89)*	28.6	1.23 (0.91~1.40)	30.4	1.17 (0.95~1.44)
	T3	36.9	2.33 (1.80~3.02)*	37.6	1.64 (1.32~2.03)*	32.1	1.28 (1.04~1.58)*
High blood sugar	T1	25.4	1 (Ref.)	20.9	1 (Ref.)	27.8	1 (Ref.)
	T2	26.6	1.13 (0.89~1.44)	24.3	1.33 (1.05~1.68)*	23.7	0.92 (0.73~1.15)
	T3	21.5	1.14 (0.85~1.49)	28.2	1.71 (1.34~2.17)*	21.9	0.74 (0.59~0.93)*
Hypertension	T1	43.6	1 (Ref.)	42.3	1 (Ref.)	50.5	1 (Ref.)
	T2	49.1	1.41 (1.15~1.74)*	44.3	1.16 (0.95~1.41)	42.3	0.78 (0.64~0.95)*
	T3	46.7	1.49 (1.17~1.90)*	53.8	1.66 (1.35~2.05)*	46.6	0.88 (0.72~1.08)
HighTG	T1	24.5	1 (Ref.)	21.5	1 (Ref.)	27.7	1 (Ref.)
	T2	28.7	1.32 (1.05~1.64)*	26.5	1.33 (1.07~1.66)*	28.0	1.16 (0.94~1.43)*
	T3	33.3	1.61 (1.25~2.08)*	38.6	2.28 (1.83~2.85)*	30.9	1.38 (1.2~1.70)*
Low HDL-C	T1	13.7	1 (Ref.)	14.6	1 (Ref.)	18.0	1 (Ref.)
	T2	17.5	1.16 (0.89~1.52)	16.7	1.05 (0.81~1.36)	18.7	1.13 (0.89~1.44)
	T3	24.8	1.40 (1.04~1.88)*	24.8	1.57 (1.22~2.00)*	19.3	1.36 (1.07~1.74)*

The Logistic regression analysis equation was adjusted for age, gender, smoking history and drinking history. % indicates the incidence of MS or its components after the tertile of THs; OR represents odds ratio; CI represents confidence interval; TI-T3 represents the 1st to 3rd quantile; * indicates P< 0.05.

## 4 Discussion

### 4.1 Epidemiological characteristics of hypothyroidism in Qinghai Province

#### 4.1.1 Prevalence of hypothyroidism in Qinghai population

With the change of people’s diet structure in modern society, the acceleration of life style and work pace, and the improvement of medical diagnosis and treatment technology, the overall prevalence of hypothyroidism is increasing year by year. In recent studies at home and abroad, it is generally believed that iodine deficiency and Hashimoto’s thyroiditis are the main causes (5), but excessive iodine can also induce hypothyroidism (6, 7). Since the implementation of the strategy of salt iodization to prevent iodine deficiency disorders in our country in 1994, the overall iodine nutritional status of residents has been improved. Some studies have suggested that the iodine nutritional level of residents in Qinghai Province has been in the state of iodine excess (3), and the prevalence of hypothyroidism, as an important part of the evaluation of iodine nutritional status, deserves attention. In this study, the prevalence of hypothyroidism and related risk factors were analyzed and discussed. The final statistical results showed that the total prevalence of hypothyroidism in adults of population in Qinghai Province was 30.25%, of which the prevalence of subclinical hypothyroidism was 29.22%. The prevalence of hypothyroidism was 1.03%, which was significantly higher than that of clinical hypothyroidism. It can be seen that hypothyroidism has become a common endocrine disease affecting nearly one in three adults in this area.

In a domestic epidemiological survey of thyroid diseases in Chengdu community population, the prevalence rates of clinical hypothyroidism and subclinical hypothyroidism were 0.96% and 5.5%, respectively (8). Peng Wen et al. (9) showed that the prevalence rates of clinical hypothyroidism and subclinical hypothyroidism were 0.32% and 4.82%, respectively, in the survey of the prevalence of thyroid diseases in Nanjing population. The prevalence of clinical hypothyroidism and subclinical hypothyroidism in this study was higher than the above results, but it was basically consistent with the data of a large study covering 10 cities in central and eastern China and the results of 31 provinces and cities in China, which showed that the prevalence of clinical hypothyroidism was 1.1% and 1.02%, respectively. The prevalence of subclinical hypothyroidism is much higher than the reported 16.7% and 12.93% (3, 10). In the studies of foreign scholars, the data of the US National Health and Nutrition Examination Survey and a screening study all showed that the prevalence of hypothyroidism (clinical and subclinical) was lower than the results of this study (11, 12). The differences in the results of the above studies are affected by various factors such as the environmental factors of different study populations, the gender and age composition of the study subjects, and the different diagnostic criteria. Qinghai Province is located in the northeast of the Tibetan Plateau, and has unique geographical environment and living habits, which can affect the prevalence of hypothyroidism in Han adults in this region. Additionally, since the iodine supplementation policy was implemented, Qinghai has been classified as an iodine-overdose area, which is related to the elevation of serum TSH levels caused by iodine. Furthermore, there is a negative correlation between the pO2 and TSH values (13), whether changes in THS, T3, and T4 values may play a role in an adaptive mechanism needs to be further verified through future relevant experiments.

In this study, the prevalence of clinical hypothyroidism, subclinical hypothyroidism, and hypothyroidism (including clinical hypothyroidism and subclinical hypothyroidism) in women were 1.42%, 35.28%, and 36.69%, respectively, which were significantly higher than those in men (0.73%, 23.56%, and 24.30%, respectively, P< 0.01). This gender difference is consistent with the research results of Bjuro T et al. (14) and Kutluturk et al. (15). The mechanism of the influence of gender on the prevalence of hypothyroidism has not been fully clarified, but as early as 1977, Lean et al. (16) found that thyroid hormone secretion can be increased under the joint action of estrogen and progesterone, which may provide a partial explanation for the higher prevalence of hypothyroidism in women than in men. In addition, the prevalence of clinical hypothyroidism was the highest in the ≥70 age group (2.63%), and the lowest in the 18-year age group (0.0%). The difference between the age groups was statistically significant (P=0.012). The prevalence of subclinical hypothyroidism was the highest in the 60-year-old group (40.68%), and the lowest in the 30-year-old group (20.66%). The difference between the age groups was statistically significant (P< 0.001). In general, the elderly are more likely to develop hypothyroidism, which is consistent with the research reports of foreign scholars (14, 17). The investigation of Peng Wen et al. (9) in China also confirmed that the prevalence of hypothyroidism increased with age. The reason for the higher prevalence of hypothyroidism in the elderly may be related to the degenerative decline of thyroid function and the relative insufficient iodine intake in the elderly, but the detailed mechanism still needs to be further studied.

#### 4.1.2 Risk factors for hypothyroidism

By comparing the prevalence of hypothyroidism with different lifestyles, it was found that the prevalence of clinical hypothyroidism, subclinical hypothyroidism and hypothyroidism in smokers and drinkers were significantly higher than those in non-smokers and non-drinkers (P< 0.05). Multiple Logistic regression analysis showed that smoking and drinking were risk factors for hypothyroidism. No correlation was found between education level, salt intake, seafood intake, household income and the prevalence of hypothyroidism. In previous studies, many reports have confirmed the close association between smoking and hypothyroidism, but the conclusions are not the same. This study is not only consistent with the results of a domestic study on the prevalence of abnormal thyroid function in Zhoushan area (18), but also consistent with the conclusions of Soldin et al. (19) abroad, both of them believe that smoking is a risk factor for hypothyroidism. Even the study of Carrillo et al. (20) also showed that second-hand smoke causes greater damage to thyroid function. This is also the reason why some non-smokers suffer from hypothyroidism. Some toxic substances can be produced when tobacco is burned, and its metabolites can cause thyroid hormone synthesis disorders, which leads to adverse effects of smoking on thyroid function. However, some studies believe that smoking is negatively correlated with hypothyroidism (21), and some studies believe that there is no correlation between the two (22). The differences in the conclusions of these studies may be related to the sample size, the evaluation methods of smoking amount and other factors. Therefore, further studies with fine classification and design of smoking duration and smoking amount are needed to further clarify the correlation between smoking and hypothyroidism. This study also found that alcohol consumption was also a risk factor for hypothyroidism, while Carle et al. (23) believed that appropriate alcohol consumption was a protective factor for autoimmune hypothyroidism. Effraimidis et al. (24) stratified alcohol consumption and found that alcohol consumption greater than 140g per week could slow down the progression of hypothyroidism, but the specific protective mechanism has not been clarified. In this study, there was no strict quantitative classification of alcohol consumption in the study population, which was only divided into drinking and non-drinking, and it was a subjective description, so the study results could be affected to a certain extent.

### 4.2 Reference range of normal thyroid hormone levels in Qinghai population

Reviewing the previous literature, this study is the first time to establish normal reference ranges of THs for the Han population in Qinghai province after strict exclusion. The 95% reference ranges of TSH, FT4, FT3 were 0.43-5.51mIU/L, 11.0-20.4pmol/L, 3.63-5.73pmol/L, respectively. The mean value of TSH in the 71-year-old group was the highest among all the age groups (P< 0.05), while FT3 and FT4 had no significant difference among different age groups (P > 0.05). The TSH level of male was lower than that of female (P=0.010), and the FT3 and FT4 levels of male were higher than those of female (P< 0.001). The reference ranges of FT3, FT4 and TSH provided by the reagent suppliers were 3.80-6.00pmol/L, 7.86-14.41pmol/L and 0.34-5.60mIU/L, respectively. The reference ranges established in this study were not consistent with those provided by reagent suppliers, and the overall reference range of TSH was slightly narrowed (0.43-5.51mIU/L vs 0.34-5.60mIU/L). The upper and lower limits of FT4 were slightly higher (11.0-20.4pmol/L vs 7.86-14.41pmol/L). This discrepancy may be related to the different methods used to define the reference range population. Both the results of the National Health and Nutrition Examination Survey (NHANES) phase III study and the National Society of Clinical Chemistry (NACB) suggest that strict exclusion criteria should be followed for the reference range population, including excluding those with positive thyroid auto-antibodies and abnormal thyroid ultrasound. Especially for those with abnormal thyroid ultrasound, most relevant studies in China did not screen, which directly makes the results difficult to be promoted and certified. However, in this study, all subjects were examined by ultrasound and excluded, and the reference value population was strictly screened. In addition, reagent suppliers have not considered and explained the iodine nutritional status. As mentioned above, the overall iodine nutritional status has changed greatly after the national implementation of iodized salt in Qinghai, which is sure to affect the THs results of the local population. One study that included both mild iodine deficiency and iodine excess areas obtained a TSH reference range of 0.3-4.8mIU/L (25), and the upper limit was slightly lower than the results of this study. However, the reference range of TSH in this study is similar to the results reported by Li et al. (48), and this study was conducted in the population in iodine sufficient areas.

The results of thyroid function indexes are directly related to the laboratory test methods used. Liquid chromatography-tandem mass spectrometry (LC-MS/MS) is currently known to be the most accurate method for detecting THs (26, 27). However, the immunodetection method was used in this study, and the sensitivity of the immunodetection method will decrease at low concentrations of THs (28, 29). Studies have found that the diagnosis of hypothyroidism based on immune detection method can cause 65% of patients to be missed, but compared with the use of LC-MS/MS method, the missed diagnosis rate can be significantly reduced (28). Due to the limitation of economic and technical conditions, it is not possible to achieve the full coverage of thyroid function detection by LC-MS/MS method in China. Therefore, it is still reasonable and necessary to establish the reference range of THs by immune detection method. Secondly, the number of 18- years old group and over 70 years old group included in this study is relatively small, which may also affect the accuracy of the results. Therefore, studies with larger samples are needed in the future, especially more studies involving young and elderly people to make up for it.

### 4.3 Cross-sectional comparison of normal thyroid function and metabolic syndrome in Qinghai population

THs plays an important role in the process of energy regulation and cell metabolism in the human body, and can play a multi-way role in glucose and lipid metabolism, protein synthesis and decomposition, and blood pressure regulation (30). The reduction of TH leads to cardiovascular risk, cognitive decline and dementia, and high mortality in patients with renal failure undergoing hemodialysis (31). Metabolic syndrome (MS), a metabolic disorder with insulin resistance as the core, has seen a rapid rise in incidence in recent years. At present, MS has not only caused a major crisis of cardiovascular disease in the United States (32), but also has a high incidence in developing and relatively economically backward countries (33).Some studies have found that both obesity and metabolic abnormalities are associated with a high risk of hypothyroidism in men (34). In previous studies, it has been proposed that patients with thyroid disease are at high risk of MS (35). SCH is significantly associated with the increased risk of MetS and four of the five components of MetS (36). Therefore, in recent years, more and more studies have analyzed and discussed the interaction mechanism between the two (30, 36–38). However, there are few reports analyzing the association between THs and the components of MS within the normal range of thyroid function. This study conducted a cross-sectional comparison of thyroid function and metabolic syndrome in Qinghai population within the normal range, and found that FT3 and FT4 were positively correlated with the occurrence of MS, and TSH level was positively correlated with some MS components and related metabolic indicators, but there was no association with MS. The above results may be related to the regulation mechanism of body weight and related metabolic indicators. In animal models (39) and human studies (40), it was found that with the increase of adipose tissue, the conversion of T4 to T3 was gradually active. Some scholars (30, 41) have found that weight loss is often accompanied by a decrease in FT3 levels, which is due to a mechanism by which the body increases resting energy expenditure and accelerates lipolysis to achieve self-regulation of metabolic homeostasis through compensatory upregulation of FT3 levels (42).

This study found that FT4 level was correlated with many metabolic parameters (43–45). However, Taneichi et al. (46) found that FT4 level was positively correlated with BMI, visceral fat area, blood pressure and FPG. Studies by Tarcin et al. (47) and Gong et al. (48) found that MS patients had higher FT4 levels than non-MS patients, which was similar to this conclusion. A variety of factors may account for this discrepancy, such as the design of the trial protocol, inclusion and exclusion criteria, statistical methods, and genetic characteristics of the population. A large number of epidemiological data have confirmed that TSH level is positively correlated with glucose and lipid metabolism parameters and body composition (49, 50). However, no positive results were found in the final analysis of the association between TSH level and MS prevalence, which was consistent with the study of Lai et al. (51). The promoting mechanism of TSH on insulin resistance may be due to its ability to induce the expression of a variety of inflammatory factors including interleukin-6 and tumor necrosis factor-α in adipocytes (52), and the inflammatory response can enhance islet resistance (53).

## 5 Problems and prospects

Qinghai is located in the plateau, with the special geographical environment of thin oxygen concentration, long sunshine time, high ultraviolet intensity and cold climate. Even if the local residents have a good adaptation to the plateau environmental factors, this adaptation is bound to be affected to a certain extent after they suffer from metabolic diseases due to their special dietary habits. Therefore, the clinical symptoms and characteristics of patients with hypothyroidism who live in a long-term hypoxic environment are different from those of MS patients at lower altitudes. Additionally, the incidence of hypothyroidism in Qinghai Province is higher than in other regions of China, especially subclinical hypothyroidism. Considering factor, since the iodine supplementation policy was implemented, Qinghai has been classified as an iodine-overdose area, which is related to the elevation of serum TSH levels caused by iodine. Furthermore, whether changes in THS, T3, and T4 values may play a role in an adaptive mechanism needs to be further verified through future relevant experiments. This study is the first large-scale epidemiological investigation of hypothyroidism in Qinghai province, which provides reference data for the prevalence and risk factors of hypothyroidism in adults in the region, and provides theoretical basis for the prevention and treatment of hypothyroidism. The reference range of serum THs levels in Qinghai is established, and it is different from that provided by reagent suppliers, which provides a valuable reference for the diagnosis of thyroid related diseases in the local population. Although the results obtained in the analysis of the relationship between THs and each component of MS were different from those reported in previous studies, they filled the gap of such research in Qinghai area, and laid a foundation for further exploring the relationship between the normal range of THs and human metabolic levels.

## Data Availability

The raw data supporting the conclusions of this article will be made available by the authors, without undue reservation.
